# Family health sheets: a vital instrument for village health workers providing comprehensive healthcare

**DOI:** 10.1186/s12913-021-07180-y

**Published:** 2021-10-22

**Authors:** Faraz Alizadeh, Aravind Addepalli, Shombit R. Chaudhuri, Annie Modesta Budongo, Immaculate Owembabazi, Gloria Fung Chaw, Sam Musominali, Gerald Paccione

**Affiliations:** 1 Boston Children’s Hospital & Doctor’s for Global Health, 300 Longwood Ave, Boston, MA 02115 USA; 2 Albert Einstein College of Medicine & Doctor’s for Global Health, 1300 Morris Park Ave, Bronx, NY 10461 USA; 3 Kisoro District Hospital & Doctors for Global Health, Kisoro District Hospital, Kisoro, Uganda; 4 Montefiore Medical Center & Doctor’s for Global Health, 111 E 210th St, Bronx, NY 10467 USA

**Keywords:** Community health workers, Village health workers, Health census, healthcare workforce, task shifting, Community health, Non-communicable diseases, Child health, Woman’s health, sanitation

## Abstract

**Introduction:**

Community Health Worker (CHW) programs have long been used to provide acute care for children and women in healthcare shortage areas, but their provision of comprehensive longitudinal care for chronic problems is rare. The Village Health Worker (VHW) program, initiated in 2007, is an example of a long standing “horizontal” CHW program in rural Southwestern Uganda that has delivered village-level care for chronic disease based on a biannual village health census that identifies individual and family health risks. To facilitate continuity of care for problems identified, health census data were electronically transformed into family-specific Family Health Sheets (FHS) in 2016 which summarize the pertinent demographic and health data for each family, as well as health topics the family would like to learn more about. The FHS, evaluated and discussed here, serves as an epidemiologically-informed “bedside” tool to help VHWs provide longitudinal care in their villages.

**Methods:**

48 VHWs in the program completed a survey on the utility of the FHS and 24 VHWs participated in small discussion groups. Responses were analyzed using both quantitative and standard conceptual content analysis models

**Results:**

46 out of 48 VHWs reported that the FHS made them a “much better VHW.” In addition to helping target interventions in child health, women’s health, and sanitation, the FHS assisted follow-up of non-communicable diseases in the community. In discussion groups, VHWs reported that the FHS helped them understand risks for future disease, facilitated earning stipends, and increased credibility and trust in the community. Limitations cited were the infrequent updates of the FHS, only biannually with the census, and the lack of cross-reference capability by health *problem*.

**Discussion:**

The FHS supports VHWs in providing longitudinal and comprehensive healthcare of chronic diseases in their villages. Limitations, potential solutions, and future directions are discussed.

**Supplementary Information:**

The online version contains supplementary material available at 10.1186/s12913-021-07180-y.

## Background

Community Health Worker (CHW) programs have provided healthcare successfully in rural communities in various low- and middle-income countries (LMICs) throughout the global south and are strongly endorsed by the WHO as a cost-effective health delivery modality [1–5]. Communities with a shortage of trained healthcare personnel can benefit from CHWs serving as primary care providers [6, 7]. Their effectiveness stems from cultural competency and an ability to translate health through the lens of local beliefs as a community insider [8].. There is currently a shift in CHW programs, as advocated by the WHO and UNICEF, from historically “vertical” programs – focusing on one health problem such as HIV, TB or maternal mortality – to a more holistic “horizontal” approach [9–11]. One such example is the integrated community-case management portfolio where CHWs are engaged in a greater range of health activities, such as the management of multiple infections and newborn conditions to further reduce under-five mortality [12]. Other horizontal CHW models, such as the Jamkhed Comprehensive Rural Health Project in India, have shown impressive improvements in morbidity, mortality and community quality of life [13].

For decades, CHW programs have focused on child and maternal mortality. However, there is a need for adult primary care services as well: in 2017, non-communicable diseases (NCDs) in adults – primarily cardiovascular diseases, cancer, and chronic respiratory disease – were responsible for 74% of deaths worldwide, with over three quarters of NCD deaths occurring in LMICs [14]. The dual constraints of provider shortages and underfunding have led to significant interest in task shifting interventions. A growing body of literature suggests that task shifting for cardiovascular disease interventions to nurses and CHWs in LMICs can improve health outcomes, and link new patients to care [5, 15–21].

In the setting of limited resources, CHW programs must develop strategies to provide care to the most at-risk families. The Andean Rural Health Care program in Bolivia developed such a model to provide comprehensive clinical and preventative services, the census-based impact-oriented (CBIO) approach [22–24]. CBIO allowed CHWs to focus their activities based on community child and maternal risk factors identified through routine home visits that helped define community health needs. Villages using the CBIO approach saw a 52% reduction in under-five mortality (an absolute difference of 107 deaths per 1000 children under age five years compared to villages without the CBIO program) [23]. CBIO’s health education focused on pneumonia and diarrhea in children, did not incorporate adults, and was not specifically targeted towards families at risk for these issues, but rather the entire community.

In this report, we introduce and evaluate the Family Health Sheet (FHS), a census-based tool that provides Village Health Workers in Kisoro, Uganda with family-specific health information to guide longitudinal primary care, particularly for chronic disease.

### Kisoro, Uganda and the village health worker program

The Kisoro district in Southwest Uganda is home to a generally poor and rural population of 287,000, 89% of whom are subsistence farmers [25]. Two hospitals serve the district – one public and one private. Uganda has a basic CHW program, the Village Health Teams, which assists with government outreaches and provide basic care [26, 27]. In 2007, Kisoro District Hospital partnered with Doctors for Global Health, a US-based NGO, and the Albert Einstein College of Medicine/Montefiore Hospital to create the Village Health Worker (VHW) program with a mission to provide a broader spectrum of primary care services in hard-to-access villages. VHWs are elected by their community members, are at least 20 years old with six years of primary education and, after a 50-day training course over 18 months, successfully certified in six domains of service: acute care, chronic disease, environmental health, child health, women’s and reproductive health. Presently there are 52 VHWs in the program, covering 50 villages, each responsible for ~ 500–1000 people [28].

VHW activities include 1) prevention– education, referral for antenatal care and family planning, counseling about disease risk and domestic violence; 2) diagnosis – of acute illness, and screening for NCDs and malnutrition; and 3) treatment – for infections, malnutrition and NCDs [29, 30]. NCD treatment by VHWs is community-based, through the Chronic Disease in the Community (CDCom) program [19, 31]. Over the years, additions have been made to the VHW program to target geriatric and mental health as well [32, 33]. VHWs are paid according to a performance-based incentive system of stipends for particular activities with high public health or clinical importance, taking into account disease incidence and effort required by VHWs [34, 35].

### Biannual health census and the family health sheets

To guide program development, a biannual “health census” was initiated in 2010. A trained project assistant partners with the local VHW to go door-to-door obtaining diverse health data: family demographics and recent changes, child health indices (e.g. immunizations and recent routine deworming), women’s health indices (e.g. cervical cancer screening, family planning), chronic disease, status of high-risk families (e.g. three children under five years, malnutrition, solitary elder, child-headed, poor sanitation), disabilities, environmental health, and interest in various health topics. During the census the VHW screens for hypertension by measuring the blood pressure of every adult older than 25 years, for tuberculosis, by inquiring about chronic cough and weight loss, and for malnutrition, by measuring mid-upper arm circumference (MUAC) of children under five.

Besides a “needs assessment” to inform the activities of the VHW program generally, the primary purpose of the census is *clinical:* to generate health data that can be used at the “bedside” to inform care of individual families. But as a clinical tool, the census form is unwieldy, and much of its data does not pertain to a particular family. Thus, a special database program was developed in Microsoft Access which converts census data into a “Family Health Sheet” (FHS) – a summary of the demographic, health and medical risk data *pertinent to that family,* as well as the health topics the family would like to learn more about. Health problems and topic choices are automatically flagged on the first section of the 2-sided FHS, and the other side captures demographic data (see Appendix 1). VHWs keep all the FHSs of their village in a hard-copy binder organized by geographical location (zone) in the village and within zones indexed alphabetically by family name. VHWs were trained to use the FHS through interactive training sessions and in-field demonstrations by their supervisors, while being incentivized by stipends to adopt the tool.

The “FHS-supervision exercise” has been particularly important in facilitating FHS adoption by the VHWs. It works like this: prior to supervision every two weeks, VHWs consult the FHS binder to identify potential interventions that could be carried out in four families with health-related problems who live in close proximity to four other families they would already be visiting with the supervisor that day. Because the FHS is organized geographically by zone, this is straightforward. Then, at the discretion of the supervisor, one of the four neighbors is actually visited during supervision and an FHS-identified action performed: e.g. checking on a malnourished child, a pregnant woman, a person with a chronic disease, or giving a relevant health talk. The above protocol is incentivized financially: 3000 USh (approximately $1 USD) for adherence, versus a penalty of 5000 USh (approximately $1.60 USD) for non-adherence. This process helps VHWs systematically navigate the VHW binder, learn to use the geographical index, and develop skills in efficient delivery of multi-faceted care.

## Methods

The objectives of this study are: 1) to define the use of the census-derived FHS by VHWs operating within a horizontal, incentivized program; 2) to evaluate the utility, benefits, and shortcomings of the FHS to the VHW providing longitudinal care in her community; 3) to elucidate the next steps for the program, building on the FHS, to improve the ability of VHWs to prevent and care for chronic disease.

To these ends, a detailed 16-question survey was developed about the VHWs’ experience using the FHS and administered to all 48 VHWs available that month, more than three years after the FHS was initially implemented. The survey was followed by VHW discussion groups to clarify and explore more thoroughly selected survey responses. The study was approved by the IRB at Albert Einstein College of Medicine and the leadership of Kisoro District Hospital.

### Survey

A 16-question survey was created by the principal investigator and two medical students working with the program for a year. Formal survey validation tools were not used however the survey was edited and approved by many VHW supervisors and local hospital leaders. Survey questions were formally translated into Rufumbira (the local language) by an experienced translator and later reviewed, edited and back translated by multiple VHW supervisors. Furthermore, analysis of the final survey results revealed consistent common themes between respondents, informally validating the survey and suggesting the survey questions were well understood.

Of the 52 VHWs in the program, 48 completed the survey at a location close to their homes, divided into small groups of six to seven (See Table 1). Four VHWs were unable to participate due to scheduling difficulties. To reduce self-reported bias, VHWs were told that the purpose of the survey was a program audit about the FHS “in order to better understand what they think about the FHS” so that “the program can make changes that help them function as VHWs”. Such periodic feedback about aspects of the program is routine. All surveys were anonymous, using deidentified numbers. It was *stressed* that the survey would be anonymous and not be used to evaluate VHW performance or effect VHW compensation. Verbal consent to participate was received from all.

**Table 1:** Demographics of VHWs

	VHWs who completed the survey *n* = 48, (%)	VHWs who participated in the discussion groups *n* = 24, (%)
**Gender**		
Male	7 (15)	5 (21)
Female	41 (85)	19 (79)
**Age**		
20–30	7 (15)	2 (8)
31–40	16 (33)	6 (25)
41–50	13 (27)	7 (29)
51–60	7 (15)	2 (8)
60–70	5 (10)	4 (17)
**Education Level**		
< Primary school, grade 4	0	0
Primary school, grade 4–5	3 (6)	0
Primary school, grade 6–7	16 (33)	6 (25)
Some Secondary School	15 (31)	7 (29)
Completed Secondary School	11 (23)	7 (29)
Tertiary education (Certificate)	3 (6)	4 (17)
**Family Status**		
Has children	48 (100)	24 (100)
Partnered/married presently	44 (92)	22 (92)
**Primary** **Vocation**		
Commercial Farmer	1 (2)	0
Subsistence farmer	47 (98)	24 (100)
**VHW Cohort**^**a**^		
1st	10 (21)	6 (25)
2nd	14 (29)	10 (42)
3rd	15 (31)	9 (38)
4th	9 (19)	4 (17)
**Number of households in a VHW's village**		
50–100	5 (10)	2 (8)
100–150	9 (19)	5 (21)
150–200	11 (23)	7 (29)
200–250	18 (38)	8 (33)
250–300	2 (4)	1 (4)
> 300	3 (6)	1 (4)

48 of 52 VHWs participated in the survey, and 24 out of 48 participated in the discussion groups.

^a^1^st^ VHW cohort began training in 2007, 2nd cohort in 2009, 3rd cohort in 2014, 4th cohort in 2017

Survey administration to each small group was facilitated by a pair of experienced program staff, and focused on collecting unambiguous, individual data. 2–3 questions were read aloud by a facilitator at one time, and questions were solicited and clarified as needed. After the VHWs completed those questions, the process was repeated. Participants were given two hours to complete the 16-question survey thoughtfully. They were encouraged to take time, inquire freely if a question was unclear, answer honestly, be open and critical, and write comments. Surveys were filled out in Rufumbira and responses were translated and entered into an excel database in English.

### Discussion groups

Following survey analysis, the evaluation team developed questions to clarify and expand selected survey responses through small-group discussions. Questions were sorted into five categories: identification of health risks, process/use of FHS as compared to other ways of knowing about community problems, perception of the FHS in the community, credibility/trust within the community through FHS use, and documentation/updating of the FHS during the course of routine VHW activities.

24 of the 48 VHWs, over half of whom were considered by program leadership to be among the most open, critical, and independent, were chosen to participate in the small group discussions. These 24 were then randomly divided into six four-person groups.

To ensure independent consideration of each question while minimizing the influence of opinion leaders during the discussion, the participating VHWs were provided their discussion questions four days prior to the scheduled group discussions and encouraged to jot down impressions and opinions.

Discussion sessions took place at two separate sites convenient to participants, each lasting 2.5 h. Groups were led by a staff nurse, an experienced discussion leader, and were observed by a member of the evaluation team and a project staff member who also interpreted. Detailed notes were taken. Conversations were not audio-recorded.

The written notes were later analyzed by the larger research team. Trends and recurring themes from the six discussion groups were then documented for further analysis.

### Analysis

Our hypothesis was that the FHS is useful and helps VHWs address the health concerns of their village. Frequencies of the survey responses were analyzed. Stochastic significance of the distribution of responses between categories were measured, where relevant, using Fisher Exact test. For free response questions, answers were grouped thematically, and analyzed via standard conceptual content analysis methods.

The written notes from the six discussion groups were examined by the evaluation team and categorized by survey theme with the objective of reaching a deeper understanding of VHW practice and the deficiencies of the FHS model in helping to manage chronic disease in the community.

## Results

### Survey

Out of 48 VHWs, 46 (96%) reported that they thought the FHS made them a “much better VHW”. In a follow up question, the three most common clarifying comments were that the FHS helps VHWs better care for their community [13 VHWs (28%)], builds VHW credibility in the community [11 VHWs (24%)], and facilitates follow-up of issues that were discovered during the census [10 VHWs (22%)]. Four other VHWs also mentioned that the FHSs help equalize their efforts with all families, without favoring their closer neighbors.

Regarding frequency of use, 44 VHWs (91%) reported that they use the FHS regularly on their own outside of the bimonthly supervision sessions. In order to ascertain the perceived purpose of the FHS, VHWs were asked: “What do you think are the main objectives of the FHS?” The top three responses included: to identify the people with diseases [28 VHWs (60%)], to better understand the demographics of the community diseases [25 VHWs (54%)] and to give relevant home talks to particular family’s diseases [22 VHWs (48%)]. 40 VHWs (83%) reported that the FHS were *very* helpful for targeting high risk households for various interventions.

When asked “what information on the FHS do you find to be the most useful in delivering health services (naming at least three categories)”, VHWs top three responses included: child health [39 VHWs (81%): 14 specifically mentioning malnutrition, 10 immunizations, and two deworming; women’s health [33 VHWs (69%): seven specifically mentioning family planning and three cervical cancer screening]; and sanitation [31VHWs (65%)]. (N.B. In our program, the census identifies persons with chronic disease, and they appear on the FHS, but there is a corollary record system kept in the FHS binder used for patients with chronic disease.)

In order to gauge familiarity with the FHS, and presumably the sections most frequently used, participants were asked to name all the sections of the FHS they could remember without looking at their FHS binder. Table 2 lists the sections of the FHS with the number (%) of VHWs that named each section. The most commonly mentioned sections were family demographics (90%), child health (75%), and sanitation (75%).

**Table 2:** Categories or items of information on the FHSs that VHWs remember

FHS Section	Number of VHWs responding ***N*** = 48, (%)
Family Member Demographics (with list of Chronic diseases)	43 (90)
Child Health	36 (75)
Sanitation (Kitchen, Latrine, Animal House)	36 (75)
Women’s Health	24 (50)
Malaria Risk Factors	20 (42)
High Risk Issues	19 (40)
Request Home Talks	14 (29)
Child Deaths	12 (25)
Births	11 (23)
Adult Deaths	8 (17)
Head of Household works outside Kisoro	7 (15)
Sanitation of Home Water System	6 (13)
Cough Screen	3 (6)
Gapfura^a^	2 (4)

^a^Gapfura refers to a locally defined febrile syndrome whose treatment is a form of mutilation of the tonsils, not condoned by local medical practitioners

**Fig. 1:**
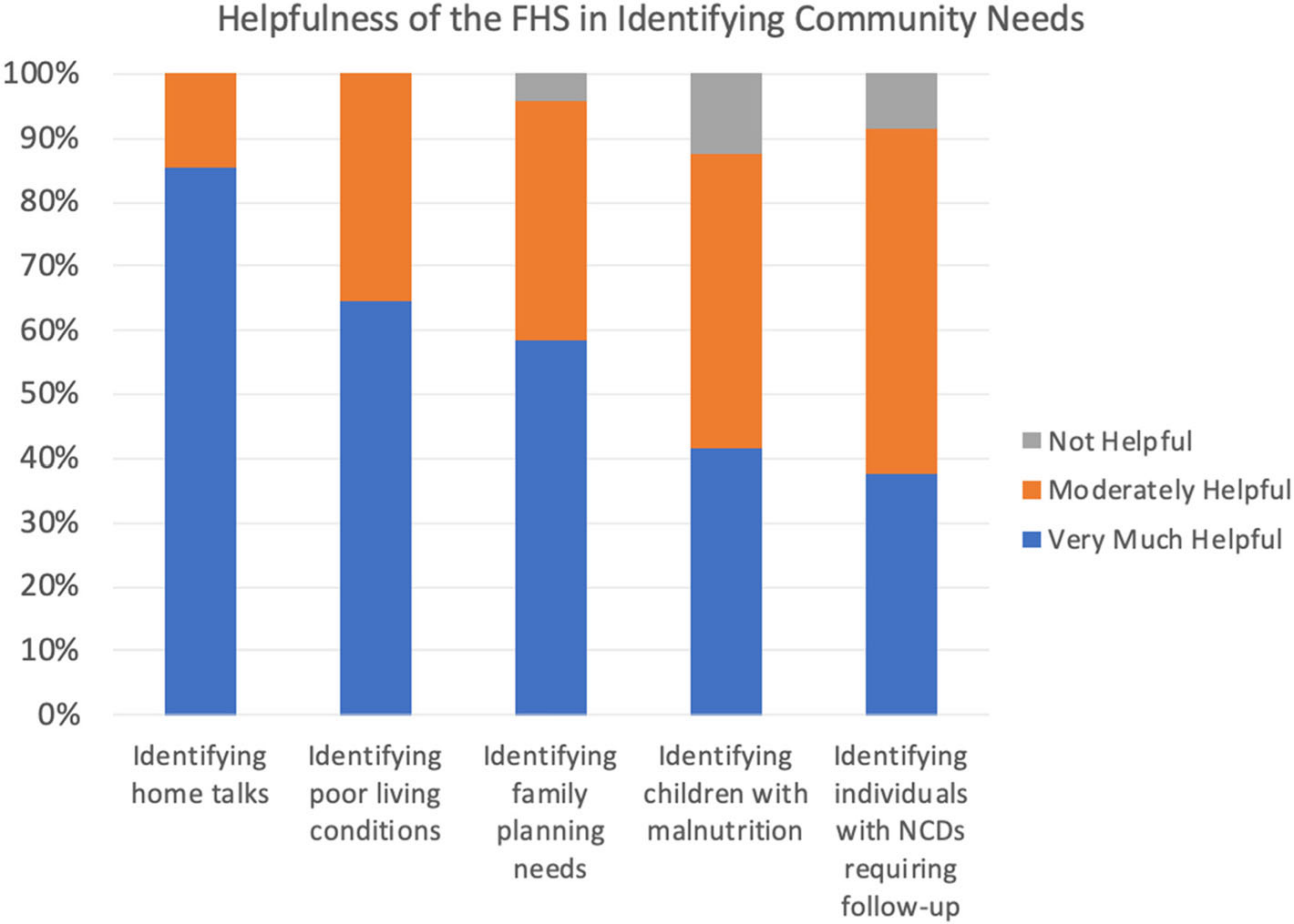
Helpfulness of the FHS in Identifying Certain Community Needs

Forty-two VHWs (83%) felt that the FHS was *very* helpful “in identifying health needs of households”, and that they “almost always use the FHS in the field”. To further assess the comparative utility of each section of the FHS in daily practice, VHWs were asked to rank on a three-point scale, “Does the FHS help you identify the following issues/problems or do the issues noted usually come to attention in a different way?” See Fig. 1 for the distribution of responses. Most VHWs reported that the FHS was *very helpful* in identifying a) families that could benefit from a home talk (85%), b) poor living conditions (65%), c) mothers who should consider family planning (58%). The FHS was also useful in identifying malnourished children (42% very, 46% moderate), and NCDs that need community follow-up (38% very, 54% moderate).

Vis-a-vis the ease of understanding the content of the FHS and the use of the physical binder and index, all VHWs reported being comfortable at the time of the survey with both FHS content and index; for about half of them, it took use in the field and, for another ~ 30%, additional tutoring, before employing them easily.

Of note, the survey had additional questions that were helpful for internal review and quality improvement but not included in this report due to space constraints and relevance to other CHW programs. All responses mentioned above were statistically significant, *p* < 0.01.

### Discussion groups

The two-hour discussions - with VHWs who had had time to reflect on the topics beforehand - highlighted both benefits and shortcomings of the FHS in VHW practice. The most important themes treated in the six discussion groups are summarized below – three positive, three negative.

#### The FHS is used to identify VHW actions appropriate for specific families

VHW work can either be *spontaneous*, e.g. when summoned for a new health problem, or *planned*, e.g. giving a home talk or following up past problems such as blood pressure in adults and MUACs in borderline malnourished children. Most days in the field are a combination of both. The FHS is used to target these latter planned activities. Most VHWs do not actually carry the FHS binder while rounding in the community but use it at home beforehand to identify pertinent activities in a particular geographical zone.

#### Risks for disease become apparent

By using the FHS to plan activities, causal relationships are more readily witnessed, understood, and incorporated into practice. One VHW commented: “I look through the FHS, see households with a certain problem, and see what things caused that condition when I visit”. The FHS assisted VHWs to conceptually associate conditions - e.g. lack of family planning and child malnutrition, or poor sanitation facilities and diarrhea/stunting - and act on those problems concurrently - e.g. checking children’s MUACs after giving a home talk on family planning.

#### The FHS facilitates earning stipends

By identifying multiple health issues that can be addressed in a given home, or in homes nearby a planned destination, the VHW can earn additional income addressing chronic health problems through the “pay-for-performance” model our program employs.

#### The FHS is not currently a “living record”

Observations or activities performed in a home are not documented in the FHS. Rather, field notes are written in random notebooks, and transferred to the stipend form for payment that month. There is no record of health or demographic observations between censuses, once the monthly stipend forms are submitted. Most VHWs wait for the next census to update their FHSs.

#### Retrieval of observations or problems from the FHS binder is tedious, and not user-friendly

While easily referencing a particular family’s health issues, the FHS cannot be used easily to find which families have particular issues of interest e.g. malnourished children, family planning, pregnancy, solitary elders, child-run families. Problems are located by navigating homes in a geographical zone page by page, relying on memory, or using the program’s designated forms to record patients with specific issues - malnutrition, chronic disease, borderline hypertension requiring follow-up, or recent inpatient ward discharge. Even the use of these “follow-up” forms, which are not meant to be long-term records, is inconsistent, challenging and prone to error.

#### Adoption of the FHS has not been uniform

While most VHWs found the FHS useful, about 10–15% did not. Whereas some VHWs were proactive, innovative, understood the function of the FHS binder, and used the FHS files to facilitate their mission and augment their stipends, for a minority, the files became a chore for supervision that otherwise sit under the bed. The discussion groups themselves, assembled to review the survey responses, created a forum to share approaches to working as VHWs, and to disseminate “best practices”.

## Discussion

The recent extensive NCD Countdown 2030 report in the Lancet highlights the challenge to human health presented by the spiraling mortality from chronic disease in LMICs [36]. Given the lack of resources in rural Africa, prevention - both primary and secondary - is key in averting both the suffering of end-stage complications of disease and the burden on families that such complications incur. In communities like rural Uganda, whose relatively few health professionals are consumed with confronting acute illness, prevention relies on “task-shifting” diagnosis and treatment of cardiovascular risk factors to lesser trained cadres of health providers [14, 15].

The sheer magnitude of addressing the chronic disease challenge requires that the role of the most available primary care providers – CHWs – evolve from a narrow “vertical” focus on single-problems to a more comprehensive, “horizontal” perspective, applying diverse skills contemporaneously to multiple symptoms and disease treatments. However, if these new skills are used only to extend care in health facilities, up to 90% of the community’s disease burden may be missed – as with hypertension [37–40]. Conversely, if intended for sporadic use, e.g. in community-based screening “outreaches”, undue stress may be placed on other links in the disease-management chain. Simple referrals to inadequately staffed, poorly stocked clinics are unlikely to control disease and infrequently applied screening skills learned by CHWs are likely to deteriorate over time.

Our Kisoro VHW program has been addressing chronic disease since 2010, equipping VHWs to diagnose, treat, and follow chronic diseases in their community before and after complications occur. However, our decade’s experience has been sobering: for a VHW to expand practice from acute care of fever or diarrhea in young children to comprehensive care of chronic and clinically silent “risk factors” in adults, *systems* must be sensible and comprehensible at every stage [31]. In our experience, successful management of chronic problems at the community level - by VHWs with a horizontal portfolio of responsibilities - requires fine coordination of the types of system supports listed in Table 3. The Family Health Sheets, now in use for four years, were designed to enable the data collected during the village’s biannual health census to serve directly the populations surveyed, rather than fill tables in periodic reports. They were to be part of the “effector arm” of a community-oriented primary care model in which public health data define health needs, which are then addressed through ongoing, continually evaluated and modified, program development - in this case, Kisoro’s multi-faceted Village Health Worker program. This study was created to better assess the value and utility of the FHS, its place in longitudinal chronic disease care at the village level, and its shortcomings.

**Table 3:** Kisoro VHW program’s response to the system requirements for community management of chronic disease

Requirement for community based, CHW management of chronic disease	Kisoro VHW program responses
Early (preclinical) diagnosis through comprehensive community Screening	Biannual health Census by dedicated trained staff partnering with VHWs
Diagnostic verification by a health professional	Supervisor verification during home visit of screen-positive people
Follow-up of “borderline” cases below treatment thresholds	VHWs in the field, targeting specified clinical indices periodically and keeping track of patients via Family Health Sheets
Community-based treatment: easy-access for high-risk patients who would not seek care otherwise, with referral/consultation when needed;	Chronic Disease in the Community (CDCom) program, ongoing 10 years; 39 monthly outdoor community clinic sites; referral pathways to district hospital established
Geriatric treatment for common disabilities of aging: diagnosis and management	Elders program: VHW-mediated screening of sight, hearing, mobility, depression, and appropriate community-based therapies
Malnutrition treatment: food supplementation when needed	Community nutrition program: VHW identification, supervisor verification, village-level food supplementation
Use of health census and ongoing documentation of family observations to improve community health	Family Health Sheets and FHS binder
Maintenance of VHW interest and investment over time: incentivize health actions financially, educationally, socially; prevent burnout	Stipends for health activities undertaken, regular meetings around educational topics, bi-monthly field supervision, community respect for achievements in providing health care

Results from the survey, corroborated by the discussion groups, is that the FHSs are of significant value to the VHWs. In particular, they help VHWs provide comprehensive “horizontal” primary health services across multiple domains of health. FHSs were felt, nearly universally, to make them “much better VHWs”, facilitating efficient planning and follow-up of diverse issues in child, women’s and environmental health, prevention, health education, and chronic disease. Although nearly all areas were mentioned, FHSs were said to be most useful in addressing child health, women’s health, and sanitation – three highly prevalent themes given a lot of emphasis in VHW training with response actions that can readily earn stipends in our pay-for-performance system. While the stipend system contributes to motivation, the FHS gives VHWs the tool to act on that motivation.

VHWs attributed other valuable, albeit less-tangible, benefits to the FHSs as well - notably increased credibility and trust in the community. When entering a home with relevant information about the family, parents were impressed with their knowledge and commitment. One VHW commented, the FHS “makes me a much better [VHW] because the community members know that what I tell them is not from my head but from the research that was done.” Furthermore, realizing relationships among various health factors on the FHS, VHWs internalize the conceptual underpinnings of longitudinal and preventive care, and what “at risk” for a future event means.

The FHS has existed for four years but did not convincingly catch on until more recently. When the FHS was introduced, most VHWs had already been practicing for 5–10 years, were fully trained and certified in multiple domains of health and were successfully negotiating the pay-for-performance system with its various documentation forms. Why take the time to adopt something new without some clear benefit - be it the satisfaction of helping others, acknowledgment by the community, making money, getting promoted, avoiding penalties, etc.?

Adoption of the FHS in VHW practice became widespread when the “FHS-supervision exercise” described earlier was introduced and incentivized through stipends or penalties (i.e. using the FHS to identify four homes with health problems in the vicinity of four other homes with planned supervisory visits). It’s likely that the financial incentive prompted facility with the FHS geographical index and identification of corollary health issues, while the apparent benefit of the supervision exercise made its implementation seem feasible. By extending the model beyond supervision, more families could be engaged, stipends augmented, and trust enhanced. VHWs also realized that education, through the home talk requests documented on the FHS, could serve as an acceptable entry point into the home. In many cases, the availability of a wide variety of scripted home talks to the VHW was the primary aid in the wider adoption of the FHS.

Despite its adoption and apparent success in facilitating longitudinal care by most VHWs, the FHS has two significant limitations regarding care of patients with chronic disease. The first derives from its implementation; the second is intrinsic to its design. Although intended to be freely annotated with current observations, supplementing the FHS wasn’t repeated, reinforced, or monitored, and space for doing so wasn’t specifically demarcated on the sheet. Healthcare documentation ends with submission of the monthly stipend forms, and most VHWs didn’t see the point of additional documentation once the stipend had been collected. Thus, the FHS, as currently implemented, fails as a continuous record of the activities of a longitudinally oriented VHW. It becomes progressively outdated and less relevant in the interim two to three years before a new one is produced. Furthermore, without ongoing documentation, the recognition of worrisome patterns of illness, concerning for underlying HIV or congenital diseases like sickle cell, is hampered.

Second, intrinsic to its design (and confirmed in the discussion groups), retrieval of problem-focused information from the FHS binder is tedious. The FHS works well with geographical or family focused initiatives, but VHWs have difficulty locating or referencing *problems* that need longitudinal attention. Inability to index individuals or families by problem, stymies the implementation of timely problem-based initiatives, referrals, educational messages, or treatments.

The shortcomings mentioned above are challenges for a CHW program whose ultimate objective is the transformation of CHW practice from repetition of simple, unidimensional tasks to the longitudinal care of a village; from ministering to toddlers with fever, to identifying and caring for adults with pre-clinical and later-stage chronic disease. Thus, moving forward, our program plans two modifications to address the shortcomings of the FHS, one in fundamental design, the other in implementation.

Vis-à-vis design, the software that transforms the village health census data into the FHS has been re-designed to sort the data by *problem,* as well as by family, to produce geographically-organized lists of families and individuals with the same health issues – e.g. malnutrition, borderline hypertension, elder or child-headed households, sanitation, family planning. The new product is a collection of such problem-oriented lists of families called the *Problem-Oriented Cross-Reference (POCR)* that occupies the first 20 sheets of a 100-sheet notebook, one POCR-notebook per village. The last 80 sheets (160 pages) of the notebook are reserved for the VHW’s *field notes.* The VHWs, familiar with the difficulty of retrieving data from the FHS by problem, and who have always jotted notes on loose sheets of paper prior to documenting their actions on the stipend form, had advocated for such a tool during the FHS discussion groups and *enthusiastically* supported the POCR-notebook as the natural accompaniment to the FHS. Unfortunately, its full implementation awaits passage of the COVID-19 pandemic which has suspended most non-essential VHW activities.

To address the lack of a “living record” of village health status, the program plans to modify how supervision is carried out once full activity resumes. As the half-day supervision is being planned, 30 min will be devoted to reviewing the VHW’s notebook, FHS, and POCR – ensuring that all three are consistent and up-to-date.

Some CHW programs are incorporating electronic documentation and messaging via smart phones into field triage and treatment protocols, as recommended in the recent WHO guidelines for CHW programs [41]. While use of such mini-computers in the field will likely be incorporated in some way into our program in the future, the expense, training, network access and maintenance of such a system is not feasible at present in our region and beyond our program’s resources. Meanwhile, significant advantages of paper files include the amount of interrelated, relevant data visible on a “field”, and the ease of flipping between families or problems in the same geographical area. The paper FHS-binder and POCR could be considered the conceptual forerunners of the software necessary for comprehensive longitudinal care of a village by a VHW, more feasibly implemented on a broader-screen tablet than a smartphone.

## Conclusion

Grassroots, community-wide efforts are essential to uncover and treat the global burden of chronic disease, and CHWs are the available providers in rural regions of the world. The utility, shortcomings, and remedies for the census-FHS model speak to the inherent complexity of employing CHWs for this new role: they highlight the importance of sensible *systems* to guide action, models to script performance, incentives to encourage adoption, tangible benefits to maintain momentum, and monitoring to ensure quality.

The FHS is an instrument of “clinical public health”, the movement championed in the Bronx by Dr. Victor Sidel, past-president of the American Public Health Association (1985) whose innovative Department of Social Medicine within an urban New York hospital “chose the option of work predominantly in health care rather than medical care, with the well rather than with the sick, with ‘people’ rather than with ‘patients’.” [42, 43] Indeed, “*clinical public health*” is a fitting description for the portfolio of responsibilities borne by a VHW whose direction starts with a village health census, and whose holistic mission incorporates early detection and prevention of disease. Thus, for our program, the FHS is an attempt to bridge the gap between public health and clinical medicine, serving as both an epidemiologic medical record, and a tool that primary care providers need for continuity of care. Its successes and limitations shed light on the next steps for innovative system development for comprehensive primary care and chronic disease management in rural Africa.

## Supplementary Information

**Additional file 1: Appendix 1**. Example of the FHS. **Appendix 2.** Survey Questions

## Data Availability

All data generated or analyzed during this study are included in this published article [and its supplementary information files].

## Abbreviations

CHW: Community Health Worker
VHW: Village Health Worker
FHS: Family Health Sheets
LMICs: Low- and middle-income countries
HIV: Human Immunodeficiency Virus
TB: Tuberculosis
NCD: Non-communicable disease
CBIO: Census-based impact-oriented
CDCom: Chronic Disease in the Community
USh: Uganda Shilling
USD: United States Dollar
MUAC: Medium Upper Arm Circumference
POCR: Problem-Oriented Cross-Reference
