# Supplementary material for: Family health sheets: a vital instrument for village health workers providing comprehensive healthcare
Source: BMC Health Serv Res. 2021 Oct 22;21:1138. doi: 10.1186/s12913-021-07180-y (PMC8530699; doi:10.1186/s12913-021-07180-y)
Supplement: Supplementary file 1 — Additional file 1: Appendix 1. Example of the FHS. Appendix 2. Survey Questions [file 12913_2021_7180_MOESM1_ESM.pdf]

Appendix 1

Example of Family Health Sheet

ID	1	5/21/2019	Family Health Sheet	Village	VHW

Key Health Issues — Ibyingenzi Mubyubuzima

CDCOm PATIENT

Zone: C

Head Household: Man 1

CHILD - NO VITAMIN A [umwana - nta Vit A]

CHILD - NO ALBENDAZOLE [umwana - nta albendazole]

DEATH - ADULT / CHILD [gupfa ku muntu mukulu/ gupfa ku'mwana]

BEDNET-ABSENT / NOT USED [inzitira mibu- tabwo/ntakubukoresha]

Chronic Disease: Hypertension

CHRONIC COUGH [ikorora idakira]

KITCHEN - B [ifumbiro B]

LATRINE - B [umusarane B]

HTN: SBP above 170 [vuura umuvuduko wamaraso guri hejuru yi 170]

POOR SANITATION [isuku ribi]

REQUESTED HOME TALKS [abashaka ibiganiro byomurugo]

HEAD OF HOUSE - WORKS OUTSIDE OF KISORO [mukuru wurugo - akolera hanze ya gisoro]

Family Members — Abo Mur'iyonzu

**Name** *Izina*	**Age** *Imyaka*	**Sex** *Igitsina*	**Relationship** *Ubumwe*	**Chronic Dz** *Indwara*	**CDCom** *CDCom*	**SBP** *SBP*	**DBP** *DBP*
Man 1	31	Male	Head		☐	115	90
Woman 1	26	Female	Wife		☐	110	70
Child 3	4	Female	Daughter		☐		
Child 2	2.5	Male	Son		☐		
Child 1	1	Female	Daughter		☐		
Grandparent 1	70	Male		**Hypertension**	☑	**180**	110
					☐		
					☐		
					☐		
					☐		

Adult Deaths — Abantu Bakuru Bapfiye

**Name** *Izina*	**Age** *Imyaka*	**Location** *Yaguye he*	**Cause** *Impanve*
**Grandparent 2**	60	Home	Fever

Child Deaths — Abana bapfuye

**Name** *Izina*	**Date** *Imisi*	**Age** *Imyaka*	**Cause** *Impanve*
**Child 4**	2017	**4**	Pneumonia

Births — Abavutse

**Name** *Izina*	**Sex** *Igitsina*	**DOB** *Igihe wavutse*	**Location** *Yaguye he*
Child 1	Male	5/1/2019	Home

Child Health — Ubuzima Bw'amwana

Child Name / *Izina Ry'umwana*	Age / *Imyaka*	Immuninzation card? / *Igipande Yakingiriweho?*	Card Complete? / *Yararangije?*	Albendazole / *Albendazole*	Vit A / *Vit A*	MUAC / *MUAC*
Child 1	1	☑	☑	☐	☐	17
Child 2	2.5	☑	☑	☐	☐	15
Child 3	4	☑	☑	☐	☐	16

Women's Health — Ubuzima Bw'abagore

Name / *Izina*	Age / *Imyaka*	Kids / *Abana*	Screen? / *Igipimo*	Screen Date / *Umusi*	FP? / *FP?*	FP Method / *Uburyo bwa FP*
Woman 1	26	3	☑	1/1/2017	☐	None
			☐		☐	
			☐		☐	

Head of Household — Mukuru W'urugo

Months Head of Household lives in Kisoro
Imisi mukuru w'urugo aba Kisoro: **2**

Gapfura — Gapfura

# of Gapfura in 12 months	*# Gapfura mumezi 12*	0
# tonsillectomy	*# abamenywe*	

Malaria Risk Factors — Ibikunze Gutera Umushwiza

Does family own bednet? *mufite ubutiimba bw'imibu* ☐

If family has a bednet and doesnt use it, why?:
Niba mubufite mutabukoresha niber'iki?

Cough Screen — Gukebera Inkorora

Name / *Izina*	Age / *Imyaka*	# weeks / *Ibyumweru*
Man 1	31	0
Woman 1	26	0
Grandparent 1	70	0

Sanitation — Isuku

Kitchen	*Ifumbiro*	B
Latrine	*Icyoroni*	B
Animal House	*Inzu Yamatungo*	Not Avail

Water — Amazi

Source	*Aho Muvoma*		Treatment	*Ubuvuzi*		Storage	*Ibikiro Ry'amazi*	
Tap	*Kutapu*	No	Boil	*kuyateka*	Yes	Jerry Can	*ikidomora*	Yes
Stream	*Uruzi*	No	Sodis	*Sodisi*	No	Uncovered	*Kudapfundikira*	No
Tank	*itanka*	Yes	None	*Ntanakimwe*	No			

High Risk Issues Haribyirarikiz

- ☐ Disabled Child *Umwana wumurema*
- ☐ Soliatry Elder *Umusaza uba wenyine*
- ☐ 3 More Child *Abanabarihasi yimyaka batatu*
- ☐ Malnourish Child *Umwana urwaye mutuku*
- ☐ Child Headed House *Urugo rukuriwe n'umwana*
- ☑ Poor Sanitation *Isuku mbi / umwanda*
- ☐ Sick *Hari Urwaye*

Requested Home Talks — Iby'ubuzima byo kwigaho wifuz

☑	Breastfeeding	*Kwonsa*	☐	Sanitation	*Ibyisuku*
☐	Cancer	*Kansa*	☐	TB	*Igitundu*
☐	Chronic diseases	*Indwara zatwibanire*	☐	Antenatal care	*Gukyebeznda*
☑	Cough	*Inkorora*	☐	Female anatomy	*Umubiri wumugore*
☑	Diarrhea	*Guhitwa*	☐	Maternal mortality	*Abagore bapfa babyara*
☐	Family planning	*Kbarira urubyaro*	☐	Mental health	*Ubuzima bw'umutwe*
☐	HIV	*Slimu*	☐	Neonatal health	*Kureberera ubuhinja*
☑	Nutrition	*Imirire*	☐	Pregnancy	*Inda*
☑	Malaria	*Umushwiza*			

Appendix 2

Family Health Sheet Survey

Family Health Sheets
VHW Survey

Survey facilitators should clarify following points with VHWs

1) Begin by repeating-reminding that we want to ask a few questions about the Family Health Sheets that the VHWs are using in order to understand what they think about them.
2) When we say Family Health Sheet, we are referring to the sheet that summarizes the health status of each individual household based on the census information that we collect in your village.
3) Clarify that this survey is ONLY to help us as a program be better at helping them as VHWs. We are NOT using these surveys to evaluate them as VHWs and their performance.
4) While we are checking to make sure every VHW has filled out the survey, the actual survey will just have a de-identified ID# and we **WILL NOT KNOW** who gives what answers. Please give us honest answers.

Note for Facilitations

1) This survey has two parts. Once you collect the first part, then you can give VHWs the second part with the example FHS.
2) Please make sure you explain each question. Go through the survey, question by question, reading the question aloud and answering any questions about the meaning of the questions with the VHWs.
3) Please have VHWs write down their thoughts. They should not share their thoughts aloud and share with other VHWs.

Family Health Sheets **ID#_____**
VHW Survey Part 1

1) **Utekereza ko ar'izihe mpamvu nkuru (nangwa intego) z'igitabo c'inyandiko z'ubuzima bwa burirugo?** (*Andika izigera ku 3 nagwa 4*) [What do you think are the main reasons (or objectives) for the FHS? [write 3-4]]

____

____

____

____

2) **Ukoresha nagwa wifashisha igitabo c'inyandiko z'ubuzima bwa burirugo ikindigihe ukuyeho ku CDCom nagwa m'ukuramburwa**; [Do you use or refer to the FHS, outside of CDCom or supervision] **_____ Yego; _____ Oya;**

Niba ari yego, ukifashisha m'ugukora iki? [If yes, for what purpose(s)?]

____

____

____

Niba ari oya, n'impamvu ki? [If No, why not?] ____

____

3) **Igitabo c'inyandiko z'ubuzima bwa burirugo kigufasha ute mu kuvumbura ibyifuzo by'ubuzima mumago ari mucyaro cyawe**? *(HITAMO KIMWE MUR'IBI HEPFO)* [How helpful are the FHS to you in identifying health needs of households in your village? *[CHECK ONE]*]

___ **Ntabwo kimfasha**, iyo ndi gutambagira mumago ntabwo nigera nkoresha igitabo c'inyandiko z'ubuzima bwa burirugo.[Not helpful, when I'm in the field I really don't use the FHSs]

___ **Kirafasha mo gakeya**, njana n'icyo gitabo nkacyifashisha rimwe narimwe, ariko ntabwo ari cyane [Somewhat helpful, I carry the binder and refer to it occasionally, but not often]

___ **Kirafasha**, njana n'icyo gitabo kuva murugo nja murundi nka cyifashisha kumisi myishi igihe ndi mugiturage [Helpful, I carry the binder from household to household and refer to it on most days when in the community]

___ **Kirafasha Cyane**, hafi burikanya, nkuze gukoresha igitabo c'inyandiko z'ubuzima bwa burirugo igihe ndi mugiturage kugira ngo kinyibutse ingorane z'ubuzima z'ayo mago mbaramiye. [Very helpful, I almost always use the FHS binder in the community]

4) **Utekereza ko igitabo c'inyandiko z'ubuzima bwa burirugo kigukora umukozi mwiza ugezweho w'ibyubuzima mucyaro**? *(TONYEZA KIMWE MUR'IBI HEPFO)* [Do you think the FHS make you a better VHW? *[CHECK ONE]*]

___ **Nta ntandukano m'uburyo ntaga ubuhereza nk'umukozi w'ibyubuzima mucyaro** [No difference in how I serve]

___ igitabo c'inyandiko z'ubuzima bwa burirugo kinkora umukozi w'ibyubuzima mucyaro **mwiza mo gake**.a *little* better

___ igitabo c'inyandiko z'ubuzima bwa burirugo kinkora umukozi w'ibyubuzima kucyaro **mwiza cyane**. *much* better

Niba ari "Nta ntandukano" ni kuki igitabo c'inyandiko z'ubuzima bwa burirugo ari ntampinduka cyagukoreye? [If "no difference", why has the FHS made no difference?]

____

____

Niba kigukoze umukozi w'ibyubuzima kucyaro "mwiza mo gake" nagwa cyane, ni muburyo ki uri mwiza? [If a *little* or *much* "better", in what way are you better?]

____

____

5) Usobanukirwa neza ute insobanuro z'amagambo atandukanye hamwe n'imibare isangwa mugitabo c'inyandiko z'ubuzima bwa burirugo *[TONYEZA KIMWE MUR'IBI HEPFO]* **ID#________**

[How well do you understand the meaning of the different items – the terms and numbers - on the FHS? *[CHECK ONE]]*

_____ **Ntanabusa,** ntabwo nsobanukirwa NAKIMWE gisangwa mugitabo c'inyandiko z'ubuzima bwa burirugo [Not really, I don't understand ANY of the items on the FHS]

_____ **Gake,** nsobanukirwamo BIMWE bike atari byinshi [A little, I understand SOME items, but not most]

_____ **Neza,** nsobanukirwa BYINSHI ariko ntabwo ari byose. [Well, I understand MOST items, but not all]

_____ **Neza cyane,** nsobanukirwa BYOSE biri mugitabo c'inyandiko z'ubuzima bwa burirugo [Very well, I understand ALL]]

6) Byakugoye bingan'iki gusobanukirwa amakuru ari mugitabo c'inyandiko z'ubuzima bwa burirugo? *[TONYEZA KIMWE MUR'IBI HEPFO]* *[*How difficult was it to understand the information on the FHS? *[CHECK ONE]]*

_____ **BYARANGOYE**: ariko ubu narasobanukiwe: byasabaga inkurikizwa nyinshi, kubyigishwaho ndetse no kugerageza kubikoresha mugiturage. [DIFFICULT: I understand now, required multiple instructions,… and use in the field.]

_____ **NTABO BYAGOYE**, kandi ntanubwo byari byoroshe: ubwambere ntabwo nasobanukiwe, ariko nyuma yo kubikoresha mugiturage, narasobanukiwe. [NOT DIFFICULT, not easy: not at first, understood after using in the field.]

____ **BYARIBYOROSHE**: nasobanukiwe ibiri mugitabo c'inyandiko z'ubuzima bwa burirugo akokanya nkimara kubisobanurirwa ubwambere. [EASY: I understood the FHS items immediately after the first session explaining it]

7) Mbesi koresha ISHAKIRO ryo mugitabo c'inyandiko z'ubuzima bwa burirugo rifite amazina naho n'agacye amago atuyemo? *[TONYEZA KIMWE MUR'IBI HEPFO]* *[*Use the FHS *binder INDEX*, locations and names?] **___Yego** **___Oya**

Niba ari "Yego" Sobanura uburyo bwose warikoreshejemo. [If "Yes", describe all the ways you have used it.]

____

____

____

Niba ari "Oya" ni kuki udakoresha ishakiro? [If "No", why do you not use it?]

____

____

8) Ni gute wumva bikuguye neza*, gukoresha ishakiro ry'i*gitabo c'inyandiko z'ubuzima bwa burirugo *mugihe uri mugituragye?* *[TONYEZA KIMWE MUR'IBI HEPFO] [*How comfortable are you with using the FHS binder INDEX in the field?*]:*

_____ Ntabwo bingwa neza, kandi nkunze kugerageza kwirinda gukoresha ishakiro. [*Not* comfortable at all, and avoid it]

_____ Ni mo gake bitagwa neza [A *little* comfortable]

_____ Bigwa neza, kandi ndarikoresha iyo bikenewe. [*Very* comfortable, and refer to it as needed]

9) N'uburyo ki byakugoye kwiga gukoresha ishakiro *ry'i*gitabo c'inyandiko z'ubuzima bwa burirugo*?* *[TONYEZA KIMWE MUR'IBI HEPFO]* [How difficult was it to learn how to use the FHS binder INDEX? *[CHECK ONE]*]

_____ **BYARANGOYE**: ariko ubu narasobanukiwe: byasabaga inkurikizwa nyinshi, kubyigishwaho ndetse no kugerageza kubikoresha mugiturage. [DIFFICULT: I understand now, required instructions, tutorials, use in field]

_____ **NTABO BYAGOYE**, kandi ntanubwo byari byoroshe: ubwambere ntabwo nasobanukiwe, ariko nyuma yo kubikoresha mugiturage, narasobanukiwe [NOT DIFFICULT, but not easy: not at first, but after using in the field]

____ **BYARIBYOROSHE:** nasobanukiwe ibiri mugitabo c'inyandiko z'ubuzima bwa burirugo akokanya nkimara kubisobanurirwa ubwambere. [EASY: I understood the index immediately after the first session explaining it;]

10) N'ibicye ki nagwa n'ibintu ki bikoze inkuru iri mugitabo c'inyandiko z'ubuzima bwa burirugo?
(*Andika byinshi byose wa kwibuka*) [What are the categories/items of info on the FHSs (list as many as you can remember)]?

a. ____
b. ____
c. ____
d. ____
e. ____
f. ____
g. ____
h. ____

Family Health Sheets ID#_______

VHW Survey Part 2 (with example FHS)

11) **N'ayahe ari makuru mugitabo c'inyandiko z'ubuzima bwa burirugo usanga afite umumaro munini mugutanga ubufasha bw'ibyubuzima? [*andika nibura ibintu 3*]** [What FHS info is most useful in delivering health services? [*name at least 3]*

a. ____

b. ____

c. ____

d. ____

12) **N'ibihe bice/amasomo mugitabo c'inyandiko z'ubuzima bwa burirugo *ubona yoroshe gusobanukirwa?* *[andika* ibicye *2-4]*** [Which areas/topics on the FHS are the easiest to understand? *[name 2-4 areas]*]

a. ____

b. ____

c. ____

d. ____

13) **N'ibihe bice/amasomo mugitabo c'inyandiko z'ubuzima bwa burirugo *ubona akomeye gusobanukirwa?* *[andika* ibicye *2-4]*** [Which areas/topics on the FHS are the hardest to understand? *[name 2-4 areas]*]

a. ____

b. ____

c. ____

d. ____

14) ***Utabaze urugo ruri kugatezo k'ubuzima rufite guhemberwa nangwa kuramburwa*, ni gute igitabo c'inyandiko z'ubuzima bwa burirugo kigufasha mugutanga (kugenera) ubufasha bw'ibyubuzima bukwiranye n'andi mago ari kugatezo ko guhura n'izongorane z'ubuzima?** *[TONYEZA KIMWE MUR'IBI HEPFO]*
[*Not counting the one high risk household necessary for stipend/validation*, how helpful are the FHSs to you in delivering (targeting) the *most appropriate* health services to *OTHER* households at high risk? *[CHECK ONE]*]

____ **Ntako kimfasha, igitabo c'inyandiko z'ubuzima ntabwo kimfasha kugenera and'imago ari kugatezo**.
[Not helpful, the FHSs do not help me target OTHER high risk households.]

____ **Kimfasha mo gake, igitabo c'inyandiko z'ubuzima bwa burirugo kimfasha kugenera amago amwe namwe ari kugatezo, ariko ntabwo gikoreshwaga cyane kuvumbura amago ari kugatezo.**
[Somewhat helpful, the FHSs help me target some high-risk households, but are not used most of the time to identify them]

____ **Kirafasha, igitabo c'inyandiko z'ubuzima kimfasha kugenera amago ari kugatezo, kandi ndagikoresha ariko ntabwo ari cyane, burigihe ndi mugiturage**
[Helpful, the FHSs help me target high risk households, and I use them most, but not all the time in the field.]

____ **Kiramfasha cyane, igitabo c'inyandiko z'ubuzima bwa burirugo kimfasha kugenera amago ari kugatezo, kandi ngikoresha hafi bur'igihe mukuvumbura andi mago ari kugatezo.** [Very helpful, used almost ALL the time to identify]

15) **Hari ubwo igitabo c'inyandiko z'ubuzima bwa burirugo kigufasha kuvumbura izi ngorane zikurikira hepfo, Nangwa har'ubundi buryo izingorane hepfo zikugeraho m'ubundi buryo butandukanye?** *[TONYEZA KIMWE MUR'IBI HEPFO KANDI UKIVUGEHO]* [Does the FHS help you identify the following issues/problems, OR do the issues noted below usually come to attention in a different way? *[CHECK ONE, AND COMMENT BELOW]*]

a) **Abana bari kugatezo ko kugira indwara y'imirire mibi** [Children at risk for malnutrition]

_____ **Yego, cyane**: abana benshi mubo na vumbuye n'aberekanywe mugitabo c'inyandiko z'ubuzima ko bari kugatezo
[Yes, very much: most of the malnourished children I newly identify were flagged as "at risk" on the FHS]

_____ **Yego, ariko ntabwo ari burigihe:** rimwe na rimwe kwifashisha igitabo c'inyandiko z'ubuzima bwa burirugo bimfasha kugenderera abana nabonye bafite indwara y'imirire mibi, ariko abenshi bo mvumburaga mbamenyaga mubundi buryo.
[Yes, but not frequently: sometimes the FHS helps target children, but most children come to attention in another way.]

_____ **Oya**, hafi abana bose bo mvumburaga bafite indwara y'imirire mibi mbamenyaga mubundi buryo. [No, for almost all..]

KIVUGEHO: Ni ubuhe bundi buryo ukoresha kuvumbura abana bafite indwara y'imirire mibi?
[COMMENT: What other ways do you use to identify malnourished children?]

____

b) **Amago afite imibereho mibi, nk'amazi mabi, icoroni kibi, nibindi nibindi.** [Families poor living conditions, water, latrines]

_____ **Yego, cyane:** Ingorane nyinshi z'amazi/isuku mfashagamo n'ibyerekanywe n'igitabo c'inyandiko z'ubuzima bwa burirugo ko ziri kugatezo. [Yes, very much: most of the water/sanitation problems I help with were flagged as "at risk" on the FHS]

_____ **Yego, ariko ntabwo ari burigihe:** rimwe na rimwe kwifashisha igitabo c'inyandiko z'ubuzima bwa burirugo bifasha mu kwibanda ku bibazo biterwa ningorane y'amazi-isuku mbi ariko abenshi bafite iyingorane mbamenya mubundi buryo. [Yes, but not frequently: sometimes helps target wat-san, but most of the problems I help with come to attention in another way.]

_____ **Oya,** hafi bose mfashaga kungorane y'amazi-isuku ribi mbamenyaga mubundi buryo. [No…. in another way.]

KIVUGEHO: Ni ubuhe bundi buryo ukoresha kuvumbura ingorane z'amazi-isuku ribi mugiturage?
[COMMENT: What *other ways* do you use to identify wat-san problems in the community?]

____

c) **Kuvumbura ibiganiro byo mumago byihariye** [Identification of specific home talks]

_____ **Yego, cyane:** ibiganiro byinshi ntanga mumago n'ibyerekanywe mugitabo c'inyandiko z'ubuzima bwa burirugo. [Yes, very much: most of the home talks I give are identified through the FHS]

_____ **Yego, ariko ntabwo ari burigihe:** ibiganiro bimwe ntanga mugago mbikura mugitabo c'inyandiko z'ubuzima bwa burirugo ariko ibyinshi ntabwo arimwo mbikura. [Yes, but not frequently: most of the talks are not]

_____ **Oya**, ntakiganiro mubyo ntanga mumago nkura mugitabo c'inyandiko z'ubuzima bwa burirugo. [No, almost none.]

KIVUGEHO: Ni ubuhe bundi buryo ukoresha kuvumbura/gutegura ibiganiro bikwiranye n'amago? [
[COMMENT: What *other ways* do you use to identify appropriate home talks?]

____

d) **Ababyeyi batari gukoresha uburyo bwo kubarira urubyaro kandi byakabaye ngombwa ko babi koresha?**
[Mothers who are not on family planning, who perhaps should be?]

_____ **Yego, cyane:** Ababyeyi benshi mvumbura ko bakwiriye kubarira urubyaro, n'aberekanywe n'igitabo c'inyandiko z'ubuzima bwa burirugo. [Yes, very much: most of the mothers I newly identify appropriate for FP were flagged on the FHS]

_____ **Yego, ariko ntabwo ari burigihe**: rimwe narimwe kwifashishsha n'igitabo c'inyandiko z'ubuzima bwa burirugo bimfasha kuvumbura ababyeyi bambwe bafite kuba bari kubarira urubyaro, ariko ababyeyi benshi nigisha nuko bakabarira urubyaro mbamenyaga mubundi buryo. [Yes, but not frequently: but most I enroll in FP come to attention in another way.]

_____ ***Oya,*** hafi ababyey bose mfasha bagatandika kubarira urubyaro mbamenyaga mubundi buryo [No, …in another way.]

KIVUGEHO: Ubundi buryo ukoresha kuvumbura abagore bakwiriye kubarira urubyaro n'ubuhe?
[COMMENT: What other ways do you use to identify mothers appropriate for FP?]

____

e) **Abarwayi basibuwe mumavuriro bakwiriwe gukurikiranwa:** [Ward discharges appropriate to follow-up]:

_____ **Yego, cyane:** abarwayi bwenshi nkurikirana basibuwe mumavuriro mugiturage mbamenyeraga kurupapura ro gukurikirana ryo mugitabo c'inyandiko z'ubuzima bwa burirugo [Yes, very much: most identified on follow-up sheet in binder]]

_____ **Yego, ariko ntabwo ari burigihe:** rimwe narimwe kwifashisha igitabo c'inyandiko z'ubuzima bwa burirugo "urupapura rwo gukurikirana abarwayi" bimfasha kwibanda kubarwayi basibuwe abo nzindukira, ariko abarwayi benshi basibuwe mbavumburaga mubundi buryo [Yes, but not frequently… most identified in another way.]

_____ **Oya,** hafi bose abarwayi basibuwe mvumbuye ko bifuza gukurikiranwa mbamenya mubundi buryo. [No, almost all the WDs I identify as needing follow-up come to attention in another way.]

KIVUGEHO: ubundi buryo ukoresha kuvumbura no kumenya abarwayi basibuwe n'ubuhe?
[COMMENT: What other ways do you use to identify and keep track of recent WDs?]

____

f) Abarwayi ba CDCom bifuza gukurikiranwa [CDCom patients needing follow-up]

_____ **Yego, cyane:** abarwayi benshi ba CDCom nkurikirana mugiturage mbabonera kurupapura rwo gukurikirana abarwayi mugitabo c'inyandiko z'ubuzima bwa burirugo. [Yes, very much: most are identified on the "follow-up sheet" in the FHS binder.]

_____ **Yego, ariko ntabwo ari cyane**: rimwe narimwe kwifashisha igitabo c'inyandiko z'ubuzima bwa burirugo "urupapura rwo gukurikirana abarwayi" bimfasha kwibanda kubarwayi ba CDCom bo mbaramira, ariko abarwayi benshi ba CDCom bavumburwaga mubundi buryo [Yes, but not frequently: sometimes, but most I follow-up are identified in another way.]

_____ **Oya, hafi bose abarwayi ba CDCom mbaramira mugiturage mbavumbura mubundi buryo.** [No, almost all the CDCom patients I follow in the community are identified in another way.]

KIVUGEHO: Ni ubuhe bundi buryo ukoresha kuvumbura no kumenya abarwayi ba CDCom?
[COMMENT: What other ways do you use to identify and keep track of CDCom patients?]

____

16) Ni gute utekereza ko abandi ba VHW bazi/basobanukirwa ibintu bikoze urupapuro rw'inyandiko y'ubuzima bwa burirugo? *[TONYEZA KIMWE MUR'IBI HEPFO]*
[How well do you think most other VHWs know/understand the FHS categories/items? *[CHECK ONE]*]

_____ Nta numwe ubisobanukirwa [No one understands the categories]
_____ Abenshi ntabwo babisobanukirwa, ariko bamwe barabisobanukirwa**.** [Most do NOT understand the categories, but some do]
_____ Abenshi barabisobanukirwa, ariko ntabwo ari bose [Most understand the categories, but not all]
_____ Bose barabisobanukirwa [Everyone understands the categories]

17) Mbesi urupapuro rw'inyandiko y'ubuzima bwa burirugo rwakongerwaho/guhindurwa kugira ngo rurusheho kugufasha mukuvumbura ingorane z'ibyuvuzima zihariye mumago
[Can the FHS be improved to help you better identify relevant health issues in families and individuals?]

_____ Yego _____ Oya

18) Hari ibitekerezo watanga byo kongerwamo? [Do you have any suggestions for change?]

____

____

____

____

____
